# Does the viral subtype influence the biennial cycle of respiratory syncytial virus?

**DOI:** 10.1186/1743-422X-6-133

**Published:** 2009-09-07

**Authors:** Gordana Mlinaric-Galinovic, Gordana Vojnovic, Jasna Cepin-Bogovic, Ana Bace, Jadranka Bozikov, Robert C Welliver, Ulrich Wahn, Ljiljana Cebalo

**Affiliations:** 1 Department of Virology, Croatian National Institute of Public Health and University Medical School of Zagreb, Rockefellerova 12, 10000 Zagreb, Croatia; 2 University Children's Hospital Zagreb, Klaiceva 8, 10000 Zagreb, Croatia; 3 University Infectious Disease Hospital in Zagreb, Mirogojska 8, 10000 Zagreb, Croatia; 4 Department of Medical Statistics, Epidemiology and Medical Informatics, A. Stampar School of Public Health, Medical School University of Zagreb, Rockefellerova 4, 10000 Zagreb, Croatia; 5 Division of Infectious Diseases, Department of Pediatrics, Women and Children's Hospital, State University of New York at Buffalo, 219 Bryant Street, Buffalo, NY 14222, USA; 6 Department of Pediatric Pneumology and Immunology, University Children's Hospital Charite of Humboldt University, Augustenburger Platz 1, 13353 Berlin, Germany

## Abstract

**Background:**

The epidemic pattern of respiratory syncytial virus (RSV) is quite different in regions of Europe (biennial epidemics in alternating cycles of approximately 9 and 15 months) than in the Western Hemisphere (annual epidemics). In order to determine if these differences are accounted for by the circulation of different RSV subtypes, we studied the prevalence of RSV subtype A and B strains in Zagreb County from 1 January 2006 to 31 December 2007.

**Results:**

RSV was identified in the nasopharyngeal secretions of 368 inpatients using direct fluorescence assays and/or by virus isolation in cell culture. The subtype of recovered strains was determined by real-time PCR. Of 368 RSV infections identified in children during this interval, subtype A virus caused 94 infections, and subtype B 270. Four patients had a dual RSV infection (subtypes A and B).

The period of study was characterized by two epidemic waves of RSV infections-one, smaller, in the spring of 2006 (peaking in March), the second, larger, in December 2006/January 2007 (peaking in January). The predominant subtype in both outbreaks was RSV subtype B. Not until November 2007 did RSV subtype A predominate, while initiating a new outbreak continuing into the following calendar year.

**Conclusion:**

Though only two calendar years were monitored, we believe that the biennial RSV cycle in Croatia occurs independently of the dominant viral subtype.

## Background

Respiratory syncytial virus (RSV) causes major outbreaks of acute respiratory infections (ARIs) in children and adults. Infections manifest themselves as mild upper respiratory tract infections (URTIs) or lower respiratory tract infections (LRTIs): bronchitis, bronchiolitis, and pneumonia [1-3]. RSV outbreaks occur, in moderate climates, in winter/early spring months. A multiannual epidemiological study of RSV infections in Croatia has shown that these infections have a repeated biennial pattern [4]. The outbreaks alternated in a predictable cycle, peaking in December/January of 1994/95, 1996/97, 1998/99, 2000/01, 2002/03, 2004/05 and 2006/07, and every March/April during 1996, 1998, 2000, 2002, 2004 and 2006 [4-6]. Thus there is a two-year RSV cycle in Croatia repeating every 23 to 25 months. After a major RSV outbreak beginning in December/January, there ensues a minor one beginning 14 to16 months later (March/April peak), followed again by a major outbreak in another eight to ten months [4]. The same pattern of RSV outbreaks was also noted in Germany, Switzerland, Finland and Sweden [7-11]. Unlike Central Europe, Great Britain experiences a monophasic, annual RSV epidemic cycle [12]. In three geographically diverse regions of the United States (New York, Tennessee, Ohio), RSV infection cycles are also monophasic and annual [13,14]. RSV has two subtypes, A and B, that are distinguished largely by differences in the viral attachment (G) protein or the nuclear (N) protein. During epidemics, either subtype A or B may predominate, or both subtypes may circulate concurrently [13,14]. For example, in studies over a seven-year interval in South America, a monophasic RSV infection cycle was noted in Brazil, with a dominant subtype A [15], while Argentina registered an alternating annual domination between subtypes A and B [16]. The aim of this paper was to determine if differences in circulating RSV subtypes accounted for the established two-year cycles in Zagreb County.

## Patients and methods

The study was conducted as part of the scientific project #0005002, approved by the ethics committees of the Croatian National Institute of Public Health (CNIPH), the University Children's Hospital Zagreb and the University Infectious Disease Hospital in Zagreb. The study period lasted from 1 January 2006 to 31 December 2007. The study included all children (from birth to 18 years) with proven RSV ARIs. The subjects all came from Zagreb County and were hospitalized in the Zagreb University Children's Hospital and Infectious Disease Hospital. They were included into the study after a written consent had been obtained from their parents or caretakers.

RSV was identified in nasopharyngeal secretions (NPS) of patients by detection with monoclonal antibodies, using a direct fluorescence assay (DFA-Light Diagnostics, Chemicon International, Inc., Temecula, CA) or/and virus isolation in cell culture (Hep-2, HeLa, MRC-5) [17,18] at the Department of Virology, CNIPH.

Molecular diagnosis was performed by Real-Time RT PCR. RNA was extracted from NPS using a spin column kit (QIAamp DNA Mini Kit; QIAGEN GmbH, Hilden). A one-step real-time PCR assay was performed for detection of viral RNA using a single-tube RT-PCR kit (TaqMan One-Step RT-PCR Master Mix Reagents Kit; Applied Biosystems, New Jersey, USA). Amplification and detection were performed with a *7500 Real Time PCR System *machine (Applied Biosystems). The N gene of RSV A and of RSV B were targeted with primers and probes (as listed below), according to van Elden et al. [19], with minor modifications of the reverse primer for RSV A. Each tube contained a 25-μl reaction mix which included 2.5 μl of isolated RNA, 0.9 μM forward primer, 0.9 μM reverse primer and 0.25 μM probe. Primers and probes for the TaqMan amplification of viral RNA from RSV A and B were:

### RSV A (N gene)

f: AGATCAACTTCTGTCATCCAGCAA

r: TGTGTTTCTGCACATCATAATTAGGA

probe: FAM-ACACCATCCAACGGAGCACAGGAGA-TAMRA.

### RSV B (N gene)

f: AAGATGCAAATCATAAATTCACAGGA

r: TGATATCCAGCATCTTTAAGTATCTTTATAGTG

probe: FAM-AGGTATGTTATATGCTATGTCCAGGTTAGGAAGGGAA-TAMRA.

Statistical analysis was performed using STATISTICA for Windows, StatSoft, Inc. (1999), Tulsa, OK, USA. Chi-squared test for proportions and Mann-Whitney U test for age were used for group comparisons; differences with probabilities <0.05 were considered to be significant.

## Results

From January 2006 to December 2007 in Zagreb County, RSV infections were proved in 368 (162 girls and 206 boys) children aged 0-18 years. Only 6 of them (1.63%) were above 5 years while the majority (315/368 or 85.6%) were 0-2 years old (Table 1). Over one half of proved RSV infections occurred in children up to 6 months of age (188/368, i.e. 51.01%). The largest number of RSV-positive patients had a clinical picture of bronchiolitis (173, 47.01%), then URTI (108, 29.35%), bronchitis (53, 14.40%), pneumonia (37, 10.05%) and croup (2, 0.54%). RSV bronchiolitis and bronchitis were mostly found among younger children (median ages were 0.25 and 0.75 years, respectively), while URTI were identified more in slightly older children (median age 1.08 years) and pneumonia was diagnosed most commonly among even older patients (median age was 2.25 years). Among RSV-positive inpatients under the age of 12 months, bronchiolitis was diagnosed in 162/254, or 63.78%.

**Table 1:** Respiratory syncytial virus infections in Croatia in 2006 and 2007 by viral subtype, age, sex and clinical syndrome

		URTI*			**Bronchiolitis**			**Pneumonia**		**Bronchitis**			**Croup**	**Total**			
		**A**	**B**	**A+B**	**A**	**B**	**A+B**	**A**	**B**	**A**	**B**	**A+B**	**B**	**A**	**B**	**A+B**	**Total**

0-6 months	M	7	12		12	64	1		3	1	8		1	20	88	1	**109**
	
	F	3	6		20	39	1	1		2	6		1	26	52	1	**79**

6-12 months	M	2	10	1	2	15			2	3	6	1		7	33	2	**42**
	
	F	2	8		3	5		1			5			6	18		**24**

1-2 yrs	M	6	11		1	3		2	4	1	7			10	25		**35**
	
	F	5	5		2	3			5	2	4			9	17		**26**

2-5 yrs	M	4	7			2		1	2	1	1			6	12		**18**
	
	F	3	8					4	10	1	3			8	21		**29**

5-10 yrs	M								1		1				2		**2**
	
	F	1	1						1					1	2		**3**

>10 yrs	F	1												1			**1**

Total	M	19	40	1	15	84	1	3	12	6	23	1	1	43	160	3	**206**
	
	F	15	28		25	47	1	6	16	5	18		1	51	110	1	**162**

**Grand Total**		**34**	**68**	**1**	**40**	**131**	**2**	**9**	**28**	**11**	**41**	**1**	**2**	**94**	**270**	**4**	**368**

*URTI-Upper respiratory tract infection

In the entire period encompassing 2006 and 2007, subtype B RSV infections were proved almost three times more frequently than group A infections (270, or 73.4%, vs. 94 patients, or 25.5%, respectively, p < 0.001, Table 1). Subjects with subtype A or subtype B infection did not differ significantly by age. The median age for RSV patients infected by subtypes A and B were 0.58 and 0.50 years, respectively (p = 0.485).

Boys were more frequently infected by subtype B than girls. That is, subtype B was the causative agent in 160 out of 203 (88.8%) infected boys and in 110 out of 161 (68.3%) infected girls (p = 0.023). Although the age distribution of inpatients infected with subtype A or B did not differ significantly (as stated above), subtype B strains appeared to infect boys under the age of 12 months more frequently than girls of the same age (Table 1). That is, 81.8% (121/148) of RSV infections occurring in males < 12 months of age were subtype B, whereas only 68.6% (70/102) RSV infections in girls of the same age were subtype B (p = 0.016). Among children above one year of age, subtype B infections accounted for 70.9% (39/55) of infections in males, and 67.8% (40/59) of infections in girls (p = 0.718). Four patients (0.01%) had double RSV infections (subtypes A and B) (Table 1); three were boys. Two infants with double RSV infections had bronchiolitis; the remaining two had bronchitis and URTI respectively.

**Figure 1:**
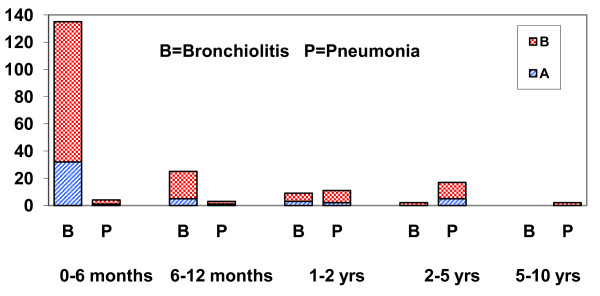
**Bronchiolitis and pneumonia (No.) caused by respiratory syncytial virus in Croatia in 2006 and 2007 by viral subtype and age**.

Bronchiolitis was caused by subtype B virus in 131/173 (75.7%) patients with this diagnosis, of whom 123 were infants (93.89%). Bronchiolitis was caused by RSV subtype A in 40 patients (23.1%), of whom 37 were infants (92.50%, Figure 1). Subtype B caused severe LRTIs (bronchiolitis and pneumonia) in 159/270 (58.9%) of those subjects with proved infections caused by this subtype. Subtype A caused bronchiolitis or pneumonia in 49/94 cases (52.1%, p = 0.25, Table 1).

**Figure 2:**
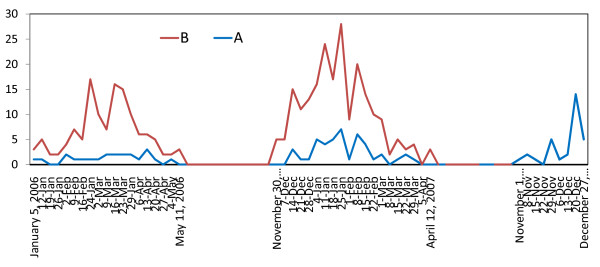
**Respiratory syncytial virus (subtypes A and B) infection occurrence (No.) by calendar week in three epidemic waves during 2006 and 2007 (1 January 2006 to 31 December 2007)**.

The year 2006 saw two epidemic waves of RSV infections. As shown in Figure 2, the first (smaller) wave began in the spring of 2006. This epidemic originated in January, peaked in March, and ended in May 2006. The second (larger) wave began in the winter of 2006/2007, starting in November 2006, peaking in January 2007, and ending in May 2007. Importantly, the predominant circulating virus in each of these outbreaks was subtype B. The ratio of RSV subtypes (A:B) was 22:105 (82.7% subtype B) for the first 2006 outbreak and 45:169 (79.0% subtype B) for the second, larger epidemic (p = 0.405); 4 patients with double infections were included in the analysis.

In the larger outbreak during 2006/2007, the subtype B wave started earlier and lasted longer than the subtype A epidemic. In the smaller spring 2006 outbreak, both subtypes began circulating at the same time, but subtype A activity terminated slightly earlier (Figure 2). In the first, smaller outbreak in 2006, the activity of subtype A was fairly constant over an interval of 14-15 weeks. In the second, larger outbreak both subtypes exhibited a more characteristic epidemic peak in January 2007 (Figure 2).

In November 2007 a new RSV epidemic began. In the first two months of this new outbreak, only subtype A virus was recovered.

## Discussion

The present study confirms the continued wintertime epidemic activity of RSV in Croatia. It also confirms the unique biennial pattern of RSV activity that is reported in Central Europe [4,7-9], but differs markedly from that observed in North and South America and Great Britain [12-16]. Many earlier studies have attempted to explain the epidemic pattern of RSV activity. Despite numerous investigations of the potential effects of climate and human behavior on RSV epidemics, no coherent explanation exists. RSV is known to exist in two subtypes, differing principally in the structure of the G or N proteins of the virus. We undertook this study to determine if the circulation of different subtypes might explain the biennial pattern of RSV epidemics in Croatia.

We found the anticipated biennial circulation of RSV in the period from January 1, 2006 through December 31, 2007 with subtype B strains of RSV predominating throughout. Subtype B accounted for 82.7% of infections in the smaller epidemic (March/April peak of 2006), and 79.0% of the cases in the larger one (December 2006/January 2007 peak). Although subtype B, in the larger outbreak, started circulating slightly earlier and its epidemic wave lasted somewhat longer than for subtype A, both RSV subtypes had the same pattern of activity in each outbreak. This occurred despite the fact subtype B infections were far more common (four times) than subtype A infections in these two outbreaks. Although we studied only a brief time interval, these findings lend doubt to the idea that subtype differences account for the existence of alternating epidemics.

In our study subtype B infection was more frequent than subtype A infection in both males and females. However, subtype B infections occurred more frequently in males less than 12 months of age than in females of the same age, while the frequency of infection with the two subtypes became equal in the two genders after 12 months of age. Our previous study of RSV activity over eleven consecutive years showed that the rate of RSV-related hospitalizations was higher in boys (59%) than in girls. However the predominance of RSV infection in males versus females was also observed among those with URTI as well as LRTI, and also among subjects with infection due to other viruses or those cases in which no virus was detected [20]. Thus we would expect the overall predominance of subtype B in males, because this subtype was the prevalent strain during the time of our study. We suspect that the equalization of RSV infection among older children is attributable to the smaller number of cases of children hospitalized after infancy. However it is known that airflows are lower in the lungs of male infants than female infants [21], so we cannot exclude an interaction of subtype B infection with male gender and congenitally lower in causing the higher rate of hospitalization for subtype B RSV infection among males during infancy.

Subtype B was not only more common overall, but also a more common causative agent of bronchiolitis and pneumonia than subtype A virus. Subtype B caused bronchiolitis and pneumonia in 58.88% cases, whereas group A caused 52.12% cases. This differs from the findings of Oliveira et al [15] in Brazil, where subtype A virus has predominated in the population for several years and more commonly caused bronchiolitis and pneumonia (54.68%) in comparison with subtype B (38.88%).

The results of the present study show that the cyclic nature of RSV epidemics in Croatia in 2006 and 2007 is identical to that of previous years [4,5]. We are now typing about 400 RSV strains that circulated in Zagreb county during 2008 to see whether subtype A (which dominated at the end of 2007; Figure 2) was the major subtype circulating through all of 2008. We already have data (yet unpublished) that the RSV epidemic in 2007/2008 peaked in the spring of 2008, while the following outbreak (2008/2009) appeared to be peaking in December 2008/January 2009. The papers of North and South American authors demonstrate that monophasic cycles occur annually in North and South America regardless of the dominant RSV subtype. This supports the claim that the monophasic or alternating patterns of RSV activity in these different countries are not determined by differences in the circulating subtypes [13,15].

It has been established that RSV outbreaks in Croatia have occurred in a two-year cycle for at least the past 15 years [4-6]. The effects of air temperature and humidity on this phenomenon were studied in northwest Croatia. Climate conditions correlated only with those RSV seasons when outbreaks peaked in December/January, and not with those outbreaks which occurred in the spring (March/April) [4]. An explanation for this variation has not been identified, although the effects of one extensive epidemic on partially immunizing infants, thereby postponing the next epidemic and reducing it in size, has been considered. Other unknown characteristics of the European mainland, such as environmental or geological features, may also be responsible.

These findings on periodicity of RSV infections forecast the beginning and end of all RSV epidemics, and are important for planning the prevention and control of RSV infections in the region [4,7-15], especially the timely supply and use of prophylactics (palivizumab). A greater understanding of the factors that determine RSV activity would make this timing even more precise. Subtype variations in circulating strains do not seem to be an important determinant of RSV activity. Hopefully, in the future another kind of prophylaxis, an effective vaccine, could diminish the need for an accurate prediction of RSV outbreak, and the great burden of RSV infections generally.

## Conclusion

Since the two-year periodicity of RSV infections in Croatia could not be related to climatic factors [4], we examined whether this epidemiological characteristic of RSV infections in Croatia could be related to a regular exchange of the two viral subtypes. However, according to current findings, it may be concluded that the predominant RSV subtype has no effect on the periodicity of RSV infections in Croatia.

## Consent

Written informed consent was obtained from the patient for publication of this work. A copy of the written consent is available for review by the Editor-in-Chief of this journal.

## Competing interests

The authors declare that they have no competing interests.

## Authors' contributions

GMG made substantial contributions to conception and design, analysis and interpretation of data; involved in drafting the manuscript, final approval of the version.

GV made substantial contributions to analysis of data; involved in drafting the manuscript.

JBC made substantial contributions to acquisition of data, analysis of data.

AB made substantial contributions to acquisition of data, analysis of data.

JB made substantial contributions to analysis and interpretation of data; involved in drafting the manuscript.

RCW made substantial contributions to conception and design, involved in revising the manuscript critically.

UW made substantial contributions to conception, involved in revising the manuscript critically.

LC made substantial contributions to acquisition of data.

All authors read and approved the final manuscript.
